# The Impact of Physical Therapy in Individuals With Benign Paroxysmal Positional Vertigo: A Case Report

**DOI:** 10.7759/cureus.62353

**Published:** 2024-06-14

**Authors:** Om C Wadhokar, Payal Shelar, Chaitanya A Kulkarni

**Affiliations:** 1 Public Health, Jawaharlal Nehru Medical College, Datta Meghe Institute of Higher Education and Research, Wardha, IND; 2 Musculoskeletal Sciences, Dr. D. Y. Patil College of Physiotherapy, Dr. D. Y. Patil Vidyapeeth, Pune, Pune, IND; 3 Community Based Rehabilitation, Dr. D. Y. Patil College of Physiotherapy, Dr. D. Y. Patil Vidyapeeth, Pune, Pune, IND; 4 Community Health Physiotherapy, Ravi Nair Physiotherapy College, Datta Meghe Institute of Higher Education and Research, Wardha, IND

**Keywords:** case report, vertigo, physical therapy, rehabilitation, bppv

## Abstract

Benign paroxysmal positional vertigo (BPPV) is a mechanical condition of the peripheral vestibular system. It is characterized by recurrent, short-lived episodes of vertigo caused by calcium carbonate crystals that get dislodged from the utricle and move into the semicircular canals. In this case report, a 33-year-old female presenting with complaints of neck pain and dizziness upon head movement was diagnosed with BPPV following a comprehensive evaluation, which included a thorough history, assessment, and investigations. The Dix-Hallpike maneuver was positive on the right side. The patient was then treated with canalith repositioning manuever (CRM) and conventional physiotherapy. There was a reduction in pain, improvement in range of motion, and reduction in the duration and frequency of vertigo. Therefore, it can be concluded that the application of CRM or Epley’s maneuver decreases the duration and frequency of vertigo and improves quality of life.

## Introduction

Vertigo is defined as an envisioned movement of either one’s body alone, such as swaying or rotation, the surroundings, or both, in the absence of physical movement. Many vestibular disorders, including Meniere's disease (MD) and benign paroxysmal positional vertigo (BPPV), are the most common causes of vertigo and dizziness. There are several different reasons why people get vertigo [1].

Peripheral or central positional/positioning nystagmus (PN) can be classified into three main categories: (i) Central PN, also known as PN I, is a condition in which the nystagmus persists throughout the duration while the head is in the precipitating posture. It typically originates from the center and is not accompanied by vertigo. (ii) Vertigo is a prominent feature of PN II, also known as BPPV, which is caused by a peripheral canal abnormality. (iii) Central PN and vertigo, also known as PN III or central paroxysmal positional vertigo (PPV), is a condition in which a central lesion results in short-lasting nystagmus and vertigo. Another name for this condition is pseudo-BPPV (p-BPPV) [2].

BPPV is a mechanical condition of the peripheral vestibular system that is characterized by recurrent, short-lived episodes of vertigo caused by calcium carbonate crystals, which get dislodged from the utricle and move into the semicircular canals (canalithiasis); occasionally, the crystals adhere to the cupula and cause it to become gravity sensitive (cupulolithiasis) [3]. The cardinal symptom is vertigo induced by quick changes in head position, such as rolling over in bed, lying down, gazing up, stooping, or sudden change in head position [4]. Cupulolithiasis can be distinguished from positional vertigo resulting from central nervous system disorders by its unique clinical features [5].

Clinical diagnosing testing involves the Dix-Hallpike maneuver, which is performed by placing the patient on the examination table so that his shoulders will rest level at the end of the table when he lies down. If the patient's medical history suggests BPPV, start with the least affected ear. In cases where laterality is not discernible, either ear can be examined first. Using both hands, hold the patient's head at an angle of 45° to one side. Throughout the procedure, instruct the patient to focus their look on a fixed point directly in front of them. Keep a watch on the patient's eyes and record any nystagmus or other abnormalities. Inform the patient that you will rapidly bring his head and torso backward on a count of three, such that their head will move over the end of the table. The patient should try to suppress blinks and maintain open eyesight at all times [6].

For BPPV in the posterior or anterior semicircular canal, use the canalith repositioning technique (CRT). First, rotate the patient's head 45° towards the affected side, in this case, the right. The affected right ear is then directed toward the ground as the patient is placed in the Dix-Hallpike posture. The head is then turned 90° to the left. It is essential to keep the patient's neck extended at a 30° angle while performing this step. At this point, the head should be 45° to the left. With their head still turned 45° to the right, the patient is gently raised up to a sitting position after being rolled onto their right shoulder. After that, the patient can be given a soft collar [7].

## Case presentation

A 33-year-old female optometrist, by occupation, was referred to the Out-Patient Department at Dr. D. Y. Patil College of Physiotherapy, Pune, India, for evaluation of dizziness while moving her head, and neck pain, which was aggravating movements and relieving rest. She works approximately eight hours per day. She reported a two-year history of neck pain, which was aggravated 15 days ago, with an intensity of 7/10, along with a two-month history of vertigo following a fenestration ear surgery (right). Activities that require performing quick movements, such as supine to sit, rolling over to the other side, or lying down, lead to a change in the orientation of the head, which further triggers vertigo lasting for approximately 30 seconds. The neck pain was believed to be present due to static posture during long working hours. The vertigo is not correlated with syncope or auditory symptoms such as hearing loss, tinnitus, and aural discomfort. The patient shared a history of fenestration ear surgery.

Clinical findings

**Figure 1:**
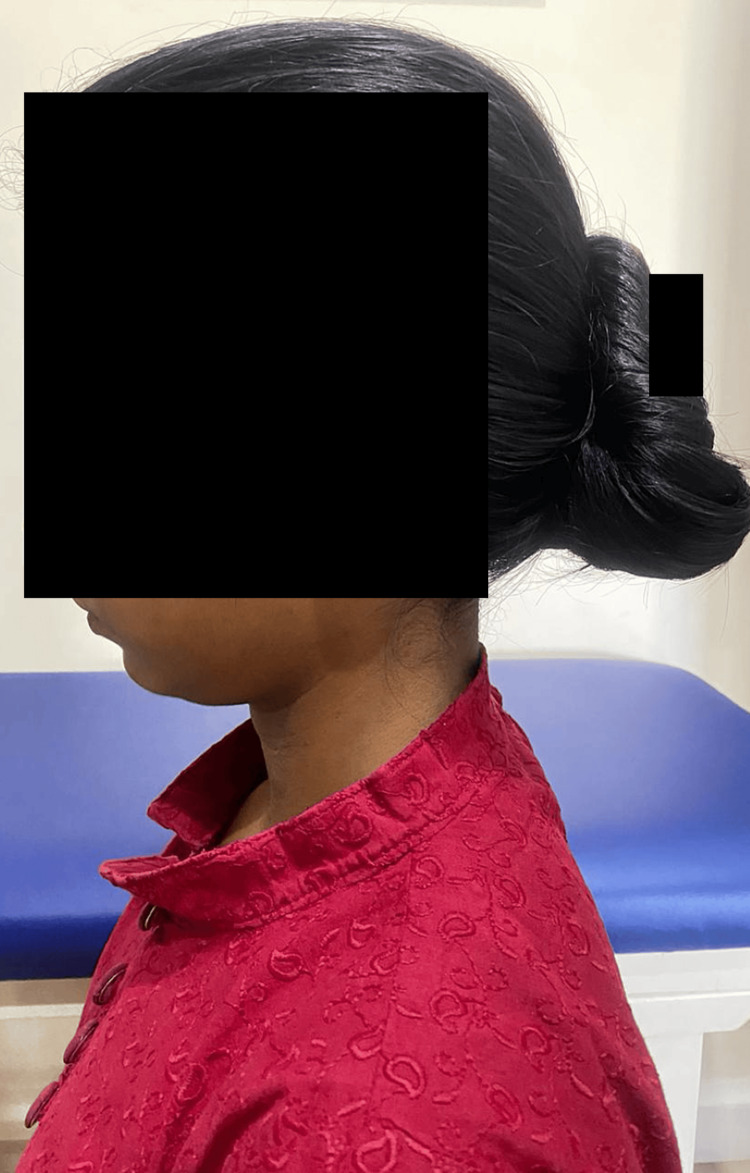
Lateral view of cervical spine

The patient's physical observation depicted reduced cervical lordosis (Figure 1).

On palpation of the left upper fiber of the trapezius, a spasm was noted. Examination revealed a reduced active cervical range of motion, characterized by painful end ranges of neck flexion, extension, and right lateral flexion, along with moderate tightness in the bilateral trapezius and levator scapulae (Table 1).

**Table 1:** Pre-treatment active range of motion

Range of motion before treatment	Degrees
Cervical flexion	0-70°
Cervical extension	0-65°
Right lateral flexion	0-30°
Left lateral flexion	0-35°
Right rotation	0-75°
Left rotation	0-75°

**Figure 2:**
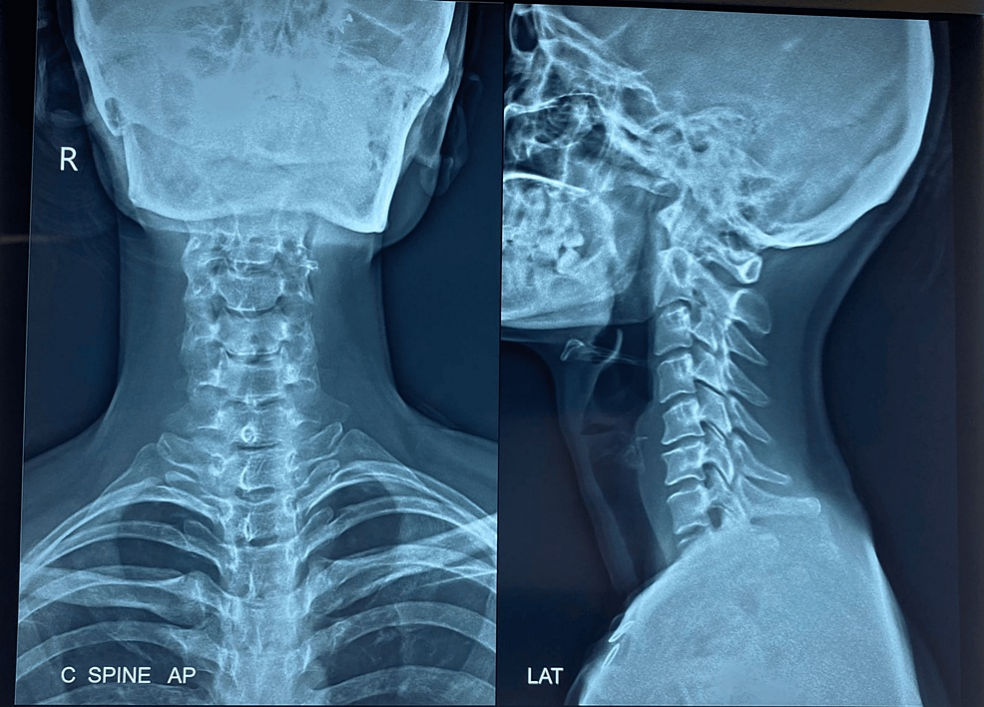
X-ray showing an anteroposterior and lateral view of the cervical spine

On physiotherapy evaluation, the patient’s Dix-Hallpike test came positive on the operated side, i.e., the right side, triggering vertigo without the occurrence of nystagmus. Weber’s and Rinnie’s tests revealed normal functioning of the vestibulocochlear nerves bilaterally. The cervical X-ray disclosed no abnormalities except reduced cervical lordosis (Figure 2).

In the timeline of September 2023, fenestration ear surgery was conducted, and she started experiencing dizziness while moving her head post-surgery in the same month. In November 2023, physiotherapy rehabilitation was initiated. Following the diagnosis, the findings were in accordance with BPPV following fenestration ear surgery. Therapeutic interventions, such as physiotherapy intervention, were initiated in November 2023, which consists of treatment for neck pain and BPPV (Table 2).

**Table 2:** Physical therapy management

Exercise protocol	Duration and frequency	Rationale
Hot fomentation	10 minutes twice daily for 5 days	To relieve trapezius spasm
Levator scapulae, trapezius passive manual stretching	3 sets with 30 seconds hold thrice a day	To release trapezius and levator scapulae spasm and muscle tightness
Interferential therapy	4 electrodes in a linear pattern for 10 minutes	To reduce pain
Epley’s maneuver	3 sets per session, 5 sessions per week, 2 weeks	To reposition the calcium carbonate (canaliths) crystals
Myofascial release	3 reps, 90 seconds, 2 sets	To release muscle spasm

**Figure 3:**
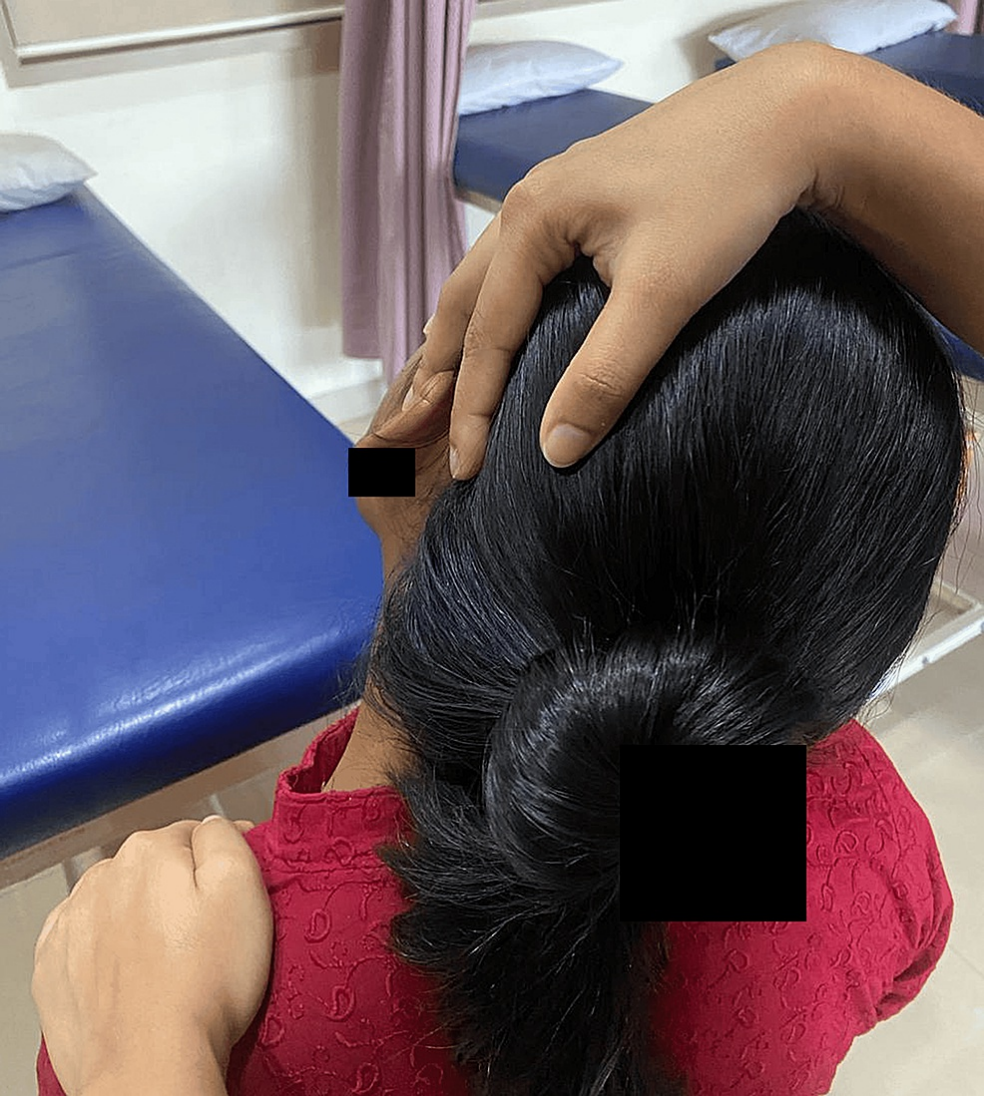
Stretching exercise for upper trapezius

**Figure 4:**
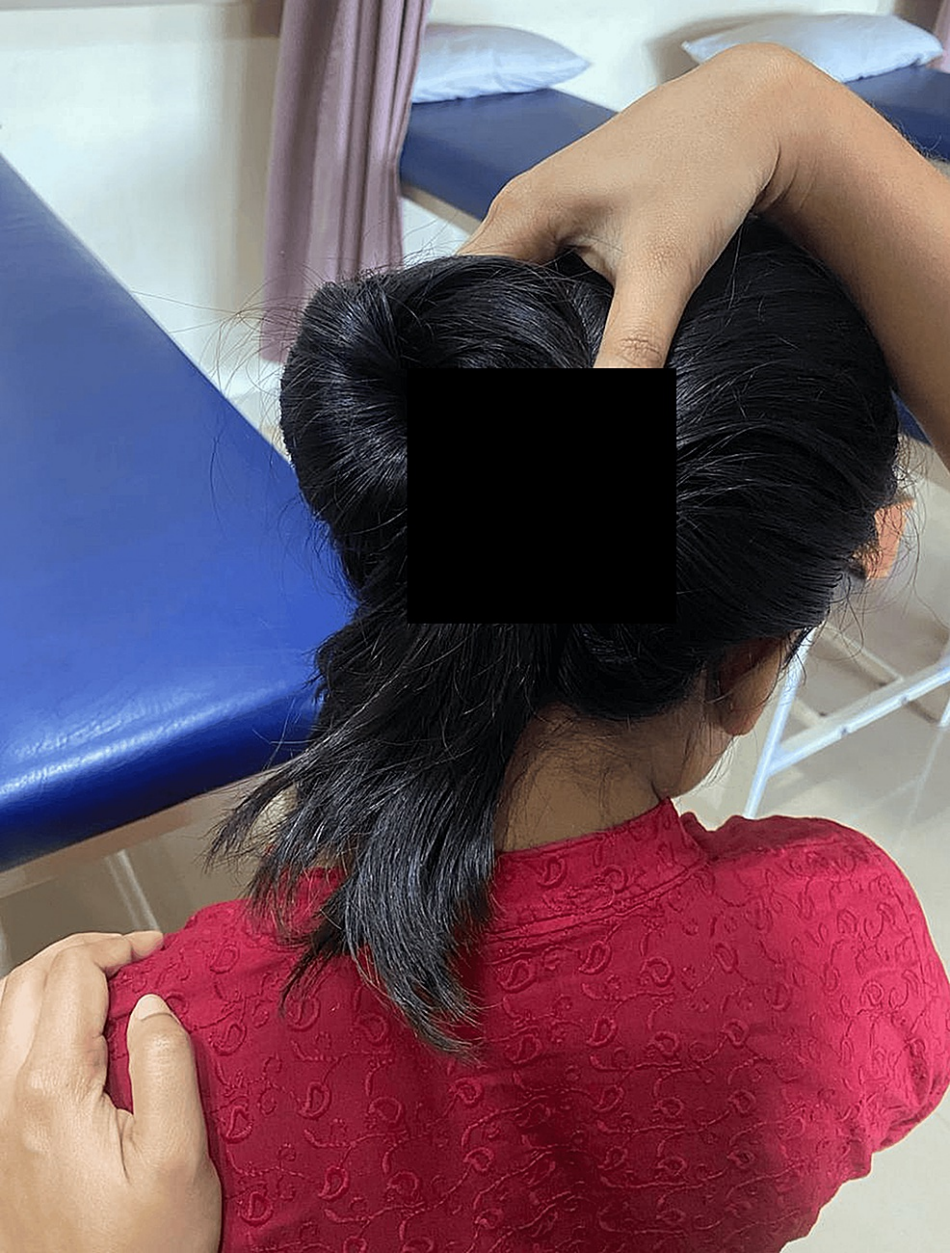
Stretching exercise for levator scapulae

**Figure 5:**
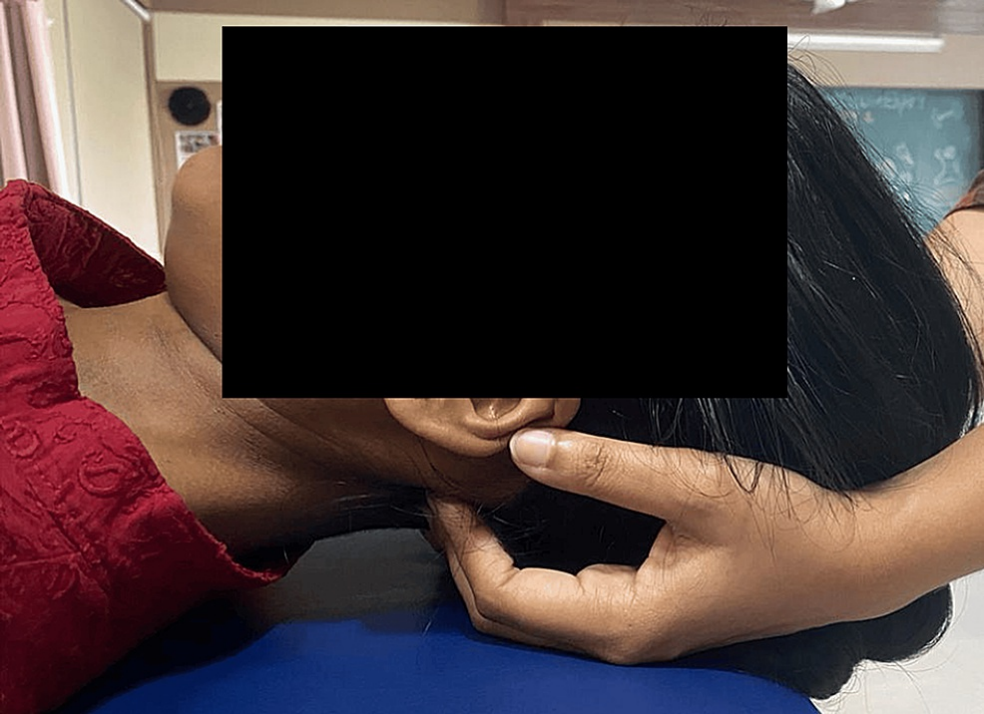
Sub occipital myofascial release

Neck physical therapy included hot fomentation, passive manual stretching, cervical stabilization exercises, and interferential therapy. Myofascial release was performed to relieve the muscle spasm (Figures 3-5).

**Figure 6:**
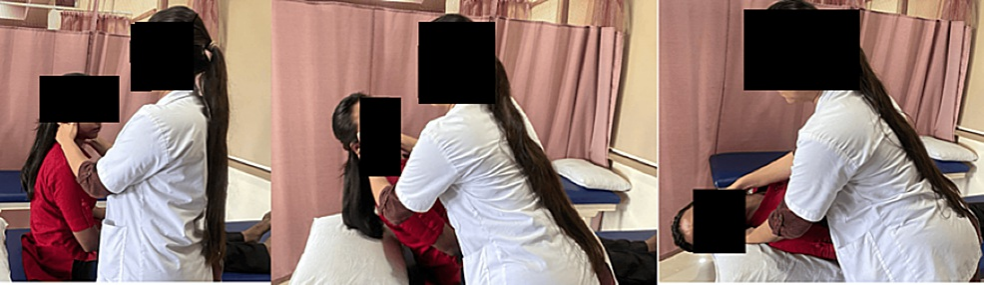
Epley's maneuver

The patient was advised to perform frequent neck movements to avoid static posture for long hours. Epley’s maneuver is shown in Figure 6.

She followed this advice and went through four sessions of physical therapy. The patient’s perspective “I took physiotherapy sessions for four days which helped me relieve my neck pain and reduce my vertigo to a certain extent.” Prior to the study, the patient was provided with an explanation, and consent was obtained.

## Discussion

BPPV is a mechanical condition of the peripheral vestibular system that is characterized by recurrent, short-lived episodes of vertigo caused by calcium carbonate crystals, which get dislodged from the utricle and move into the semicircular canals. Occasionally, the crystals adhere to the cupula and cause it to become gravity-sensitive [2,8]. Hence in order to reduce the symptoms it is essential to reposition the calcium carbonate crystals to their position [9].

Canalith repositioning maneuver (CRM) also known as Epley’s maneuver aims to alleviate the underlying physical pathology without exhausting the individual as vestibular habituation exercises do. By timing and co-ordinating precise head and body motions, these procedures aim to cause the particles to migrate from the posterior semicircular canal, across the common crus into the utricle [5,10].

There is moderately strong evidence that p-BPPV patients benefit from repeating Epley's technique. Patients who had several maneuvers in a single session accomplished better results than those who only underwent a single maneuver [11]. The present study discovered there was a reduction in the duration and frequency of vertigo experienced by the patient with each maneuver. Studies employing numerous maneuvers in each session have indicated a larger proportion of successful results, which suggests that multiple maneuvers per session may expedite the removal of debris from the semicircular canal [11]. Also, there was a reduction in neck pain to 2/10 intensity, which further improved the active cervical range of motion and the end feel was firm for all cervical range of motions (Table 3).

**Table 3:** Post-treatment active range of motion

Range of motion after treatment	Degrees
Cervical flexion	0-85°
Cervical extension	0-75°
Right lateral flexion	0-40°
Left lateral flexion	0-40°
Right rotation	0-80°
Left rotation	0-80°

## Conclusions

We conclude that individuals suffering from BPPV, along with neck pain, benefit from the application of CRM, which helps reduce the frequency and duration of BPPV. Additionally, electrotherapy application has helped individuals reduce pain and improve the overall well-being of those suffering from BPPV.
